# Androgens Profile in Blood Serum and Follicular Fluid of Women With Poor Ovarian Response During Controlled Ovarian Stimulation Reveals Differences Amongst POSEIDON Stratification Groups: A Pilot Study

**DOI:** 10.3389/fendo.2019.00458

**Published:** 2019-07-16

**Authors:** Ariel Fuentes, Karina Sequeira, Alejandro Tapia-Pizarro, Alex Muñoz, Abril Salinas, Pablo Céspedes, Javier Escalona, Ana Godoy

**Affiliations:** Faculty of Medicine, Hospital San Borja-Arriarán, Institute of Maternal and Child Research (IDIMI), Universidad de Chile, Santiago, Chile

**Keywords:** androgens, DHEA-S, poor ovarian response, follicular fluid, POSEIDON

## Abstract

Patients with poor ovarian response (POR) to exogenous gonadotropins stimulation for assisted reproductive technology (ART) have decreased circulating androgens during spontaneous cycles. The Patient-Oriented Strategies Encompassing Individualized Oocyte Number (POSEIDON) is a 4-tier stratification of women with POR to controlled ovarian stimulation (COH) based on age and biomarkers of ovarian reserve has been proposed to maximize the clinical management of this group for ART. The aim of the present study was to characterize the levels of androgens during COH in follicular fluid (FF) and serum in POSEIDON subgroups and compared them with women of normal ovarian response. Sixty nine consecutive patients undergoing ART were included and testosterone, androstenedione, dehydroepiandrosterone sulfate (DHEA-S), estradiol, sex hormone-binding globulin (SHBG), and insulin-like growth factor 1 (IGF-1) were measured in serum and FF collected at the time of oocyte pick-up. The number of retrieved oocytes was registered for each patient for their allocation to the respective POSEIDON subgroup. The control group comprised 19 women and the POSEIDON group 1 (age < 35, normal ovarian reserve biomarkers) *n* = 14, group 2 (age ≥ 35, normal ovarian reserve biomarkers) *n* = 8, group 3 (age < 35, poor ovarian reserve biomarkers) *n* = 6 and group 4 (age ≥ 35, poor ovarian reserve biomarkers) *n* = 22. Serum levels of total testosterone, androstenedione and DHEA-S were not different in group 1 vs. control but significantly decreased in group 3 vs. control. DHEA-S in FF was also significantly decreased in group 3 vs. control. In addition, serum testosterone was decreased in groups 2 and 4 vs. control; and serum androstenedione and estradiol were reduced in group 4 vs. control. No differences were observed for estradiol, SHBG and IGF-1 in FF. Finally, a high correlation between serum and FF DHEA-S was observed when data from samples of all groups were pooled. Group 1 did not show hypoandrogenemia however group 3 had low levels of all measured androgens in serum and DHEA-S in FF. Such differences might help to better characterize and/or improve the clinical management of women with POR according to their respective POSEIDON stratification.

## Introduction

Controlled ovarian hyperstimulation (COH) is a key factor predicting reproductive outcomes in Assisted Reproductive Technology (ART), since the simultaneous development of multiple follicles increases the chances of transferring embryos with the highest potential to progress to a successful pregnancy. Although major progress has been achieved in ART, around 20% of women present an insufficient response to gonadotropins administered during COH (1), which have been classified as patients with impaired or poor ovarian response (POR). The Bologna criteria (2) emerged to standardize the definition of POR and have been useful in predicting the outcome of ART and for counseling purposes. However, its relevance in clinical trials has been questioned because it encompasses a large heterogeneous population that differs significantly in biologic characteristics but have in common, a reduced number of oocytes that can be obtained, with consequent poor results in assisted reproductive technology cycles (3). The POR classification further complicates the clinical management in ART since its prognosis is widely variable and depends on parameters such as number of retrieved oocytes and age (4). Several intervention strategies have been tested for improving the results in women with POR; however, the respective randomized control trials as well as the derived meta-analyses from these studies present inconsistent results (5).

**Figure 1:**
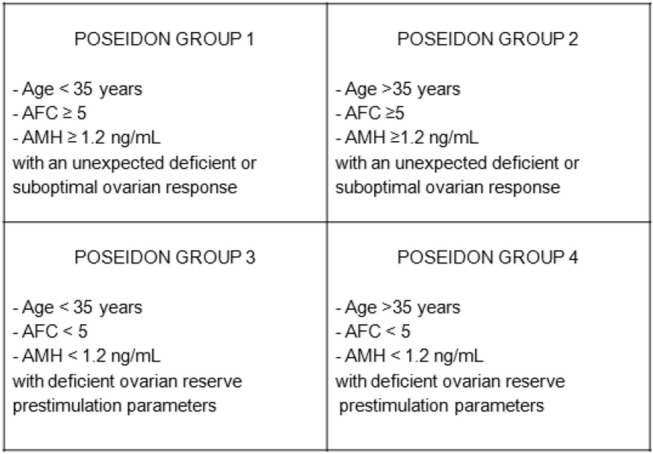
Characteristics of the 4 groups comprising the POSEIDON stratification of patients with poor ovarian response in assisted reproductive technology. AFC, antral follicle count; AMH, anti-Müllerian hormone; COH, controlled ovarian hyperstimulation. Modified from Humaidan et al. (6).

The novel POSEIDON (Patient-Oriented Strategies Encompassing IndividualizeD Oocyte Number) criteria has been suggested for identification of more homogeneous populations within POR patients to improve clinical management with tailor-made strategies that yield the best ART outcomes for each subset of patients (6). Briefly, four subgroups have been suggested based on quantitative and qualitative parameters, namely, (i) Age and the expected aneuploidy rate, (ii) Ovarian biomarkers (i.e., antral follicle count [AFC] and anti-Müllerian hormone [AMH]), and (iii) Ovarian response—provided a previous stimulation cycle was performed. Details on the characteristics of each POSEIDON group are presented in Figure 1.

Sex steroids are known to play an important role in follicular growth and development. Progestins, androgens, and estrogens are produced *de novo* from cholesterol, and synthesized by the ovary in a sequential manner (7). It has been suggested that androgens may promote folliculogenesis and potentiate the effects of gonadotropins (8). Furthermore, androgens facilitate the response of follicles to follicle-stimulating hormone (FSH) and regulate the components of the ovarian insulin-like growth factor 1 (IGF-1) signaling system. Testosterone or dehydrotestosterone increase the transcript levels of IGF-1 and IGF-1 receptors in primate ovaries (9). The presence of IGF-1 and IGF-1 receptor mRNA in granulosa cells, theca, oocytes and interstitial cells along with the fact that IGF-1 suppresses follicular apoptosis suggests that androgens may play wide range of roles in ovarian function through IGF-1, with a direct impact on improving the quality of oocytes and produced embryos (10).

Follicular fluid (FF) provides a critical microenvironment for oocyte development. FF is a transudate of serum components and the secretions of theca and granulosa cells. The study of key regulatory factors in FF may predict the physiological status of the maturing oocyte, which is important for determining the quality of the oocyte and its subsequent potential to become a viable zygote that adequately supports embryo development. Despite the fact that androgens seem to play a major role in ovarian function, their concentration in FF in poor ovarian response cases has been poorly studied. Santos *et al*. reported similar concentrations of testosterone and androstenedione in pre-ovulatory FF of POR cases and controls in unstimulated cycles (11). However, the circulating and FF levels of androgens and other molecules relevant for follicular development in POR cases during COH have not yet been determined.

A better characterization of the subgroups within the POSEIDON criteria, will allow the design of improved clinical management in each category, and will maximize the outcomes of ART. The objective of the present study was to compare the levels of testosterone, androstenedione, estradiol, sex hormone-binding globulin (SHBG), dehydroepiandrosterone sulfate (DHEA-S), and insulin-like growth factor 1 (IGF-1), in FF and blood serum of women during COH with normal ovarian response and POR stratified according to the POSEIDON subgroups.

## Materials and Methods

### Study Design

In this prospective cohort study, we evaluated patients with indication of ART at the Maternal and Child Research Institute (IDIMI), University of Chile fertility clinic (Santiago, Chile) between October 2014 and October 2017. Signed informed consent was obtained from all patients enrolled in the study under the approval of the Review Board from Servicio de Salud Metropolitano Central, Santiago, Chile (September 2013); in accordance with the Declaration of Helsinki. Recruited women had between 23 and 43 years of age and a body mass index (BMI) between 18 and 35. Additional demographic and other related clinical data were compiled from medical records and are presented in Table 1. The criteria for determining low ovarian reserve were total AFC <5 on cycle day 2 or 3 of the previous cycle to COH and a serum AMH <1.2 ng/mL. All of them underwent a comprehensive standard fertility workup including medical history, physical examination, laboratory evaluation (including hormonal profile, complete blood count, biochemistry and serology) pelvic ultrasound, a sonohysterogram to evaluate uterine cavity and tubal patency prior to initiation of any treatment. Further tests were performed for each patient and the respective partner on a case-by-case basis. Patients with endocrine disorders or anatomical abnormalities, including polycystic ovarian syndrome (PCOS), ovarian surgery, abnormal thyroid function, hyperprolactinaemia, hyperandrogenaemia as well as uterine malformation, submucous myoma, and multiple myomata were excluded.

**Table 1:** Demographic data.

	**Poseidon 1**	**Poseidon 2**	**Poseidon 3**	**Poseidon 4**	**Control**	**ANOVA**
	**(*n* = 14)**	**(*n* = 8)**	**(*n* = 6)**	**(*n* = 22)**	**(*n* = 19)**	***P***
Age (years)	32.28 ± 0.85^b, d^	39.00 ± 0.89^a, c^	31.7 ± 0.80^d^	37.8 ± 0.46^a^	32.8 ± 1.19	0.000
	(27 min−34 max)	(36 min−43 max)	(29 min−34 max)	(35 min−42 max)	(23 min−39 max)	
BMI (Kg/cm^2^)	24.84 ± 1.07	24.62 ± 1.33	24.3 ± 0.92	25.73 ± 0.98	25.9 ± 0.79	NS
Day 3 FSH (mIU/mL)	9.07 ± 2.30	7.05 ± 1.45	9.47 ± 1.96	10.64 ± 1.93	6.85 ± 1.76	NS
Day 3 Estradiol (pg/mL)	57.31 ± 12.23	45.25 ± 11.30	159.50 ± 152.50	56.67 ± 18.55	64.25 ± 34.17	NS
AMH (ng/mL)	1.21 ± 0.23	1.50 ± 0.61	0,29 ± 0.11	0.33 ± 0.05^a^	2.49 ± 0.72	0.005
AFC (both ovaries)	7.78 ± 0.71^a^	7.5 ± 0.98^a^	4.3 ± 0.33^a^	4.77 ± 0.34^a^	12,47 ± 1.73	0.005
Retrieved oocytes	4.57 ± 0.89^a^	5.25 ± 1.06^a^	2.00 ± 0.26^a^	2.45 ± 0.42^a^	13.37 ± 0.76	0.000
FSH total dose (UI)	2,971 ± 254	3,646 ± 299	3,745 ± 362	3,889 ± 316^a^	2,366 ± 230	0.001
FORT	0.55 ± 0.091^a^	0.65 ± 0.078^a^	0.47 ± 0.062^a^	0.69 ± 0.154^a^	1.42 ± 0.216	0.000

*Values are expressed as mean ± SEM. BMI, body mass index*.

a *p < 0.05 vs. Control*.

b *p < 0.05 vs. Poseidon 2*.

c *p < 0.05 vs. Poseidon 3*.

d *p < 0.05 vs. Poseidon 4*.

*Statistical significance using ANOVA withTukey post-hoc test*.

Sixty nine women were consecutively recruited between October 2014 and October 2017 that were divided into 2 categories: normal ovarian reserve (*n* = 41) and low ovarian reserve (*n* = 28) according to AMH and AFC (determined in a previous menstrual cycle) until the completion of their first cycle of ART. AFC was performed by vaginal ultrasound as part of routine functional exploration of patients undergoing ART at our center. Once they completed the COH cycle, they were assigned to one of the 4 POSEIDON groups described above or to the control group. Women that developed ovarian hyperstimulation syndrome during the ART cycle under study were excluded from the study. The follicular output rate (FORT) was calculated as the ratio between pre-ovulatory follicle count (follicles measuring >15 mm in diameter on the day of hCG administration) × 100/AFC (all follicles measuring 3–8 mm in diameter at baseline).

### COH Protocol

Participants received individualized daily subcutaneous doses of FSH (Follitropin alfa Gonal-F, Merck Serono, Germany) and HMG (Menopur, Ferring Pharmaceuticals, Parsippany, NJ, USA) at doses of 150–300 IU and 75–300 IU, respectively, for ovarian stimulation. A fixed gonadotropin-releasing hormone antagonist protocol was used with 0.25 mg of cetrorelix (Cetrotide, Merck, Rockland, MA, USA) administered subcutaneously from day 6 of gonadotropins stimulation. Final oocyte maturation was triggered when the leading follicles >17 mm with recombinant human chorionic gonadotropin (6,500 IU; Ovidrel, Merck, Rockland, MA, USA). Transvaginal ultrasound-guided oocyte pick-up was performed 35–36 h later. The number and maturity status of collected oocytes was registered. For our study it was considered only the oocytes in meiosis II stage.

### Sample Collection

Two samples of peripheral blood were collected by venipuncture. The first one was collected on day 3 ± 2 of the menstrual cycle prior to the IVF procedure, to measure baseline levels of FSH, estradiol, and AMH. The second sample was obtained on the day of oocyte pick-up, to measure androgen, SHBG, and IGF-1. Peripheral blood was centrifuged at 1,000 g for 5 min. The serum was collected and frozen at −70°C until processed in a single assay for each analyte. Thus, none of the samples were frozen and thawed repeatedly.

Undiluted FF was obtained at the time of the oocyte pick-up only from the first aspirated follicle to avoid contamination with blood, washing medium or FF from other follicles. The selected follicle was the largest (at least 15 mm) and most accessible one. Follicle size was designated based on a 70% chance of obtaining a metaphase II oocyte from a follicle >15 mm in diameter (12). The obtained FF was transferred into sterile polypropylene tubes and centrifuged at 500 rpm for 10 min to separate the FF from the debris and granulosa cells. The supernatant fluid was transferred to sterile 1.5 mL polypropylene tubes immediately, and stored at −70°C until processing for analyte measurements. The assay methods for analyte measurements are presented below.

### Assays

The levels of testosterone, androstenedione, DHEA-S, estradiol, sex hormone-binding globulin (SHBG), and insulin-like growth factor 1 (IGF-1) were evaluated in blood serum and FF. Liquid chromatography-tandem mass spectrometry (LC-MS/MS) analysis was used for testosterone, androstenedione, and DHEA-S quantifications using an HPLC Agilent 1260 (Santa Clara, CA, USA) coupled to a AB Sciex 3200 Quantum Ultra triple quadrupole mass spectrometer (Foster City, CA, USA). Samples, calibrators, and quality controls were run in duplicate and prepared according to the manufacturer's instructions. The respective sensitivities for testosterone, androstenedione, and DHEA-S were 0.01, 0.03, and 75 ng/ml; and the intra- and inter-assays coefficients of variation were 1.5, 1.2, 2.9% and 2.8, 2.5, 5.0%, respectively. Commercial radioimmunoassays (RIA) were used to analyze IGF-1 (DIAsource, Louvain-la-Neuve, Belgium), estradiol (Pantex, Santa Monica, CA, USA), and SHBG (IZOTOP RIA, Codolet, France. AMH was measured by an UltraSensitive AMH/MIS ELISA (Ansh Labs, Webster, TX, USA) with a sensitivity of 0.023 ng/mL and intra- and inter-assay coefficients of variation of 3.21 and 5.2%, respectively.

### Data Analysis

Considering a standard deviation of testosterone in FF of 563 ng/dl and a difference between the means of 480 ng/dl (13), with an α of 0.05 and a power of 80% (β = 0.20), the minimum sample size was 22 patients per group. Considering that this study has 5 groups to be compared, the total number of subjects required would be 110. Having recruited 63% of the cases, we report our results as part of a pilot study.

One-way ANOVA test was performed to assess differences in means amongst groups followed by Tukey's multiple comparison test. In relation to hypothesis contrast testing, an α threshold value of 0.05 was considered for rejecting/accepting the null hypothesis (no differences in analyte levels between groups). Pearson's correlation coefficients were used to assess the associations of serum and FF level of analytes in all samples. All statistical analyses were carried out using the SPSS® version 21.0 statistical package (SPPS Inc., Chicago, IL, USA). Data have been presented as mean ± standard error of mean, with 95% confidence intervals.

## Results

A total of 69 women were included in the study having either normal (*n* = 41) or low ovarian reserve (*n* = 28) according to AMH levels and AFC. Thus, according to the ART results, 14 women were assigned to group 1 (under 35 years of age with normal ovarian reserve and poor response to COH), 8 were assigned to group 2 (35 or more years old with normal ovarian reserve and poor response to COH), 6 of the women corresponded to group 3 (under 35 years with low ovarian reserve and poor response to COH), 22 women were assigned to group 4 (35 or more years old with low ovarian reserve and poor response to COH), and finally, 19 women were included in the control group (women with normal ovarian reserve and adequate response to COH). As expected, subjects in groups 2 and 4 of the POSEIDON classification were significantly older than those in groups 1 and 3, and the control group (39.00 ± 0.89 and 37.8 ± 0.46 vs. 32.28 ± 0.85, 31.7 ± 0.80 and 32.8 ± 1.19, respectively, Table 1). With regard to circulating AMH levels, group 4 had significantly lower serum concentrations than the control group (0.33 ± 0.05 vs. 2.49 ± 0.72 respectively, Table 1). In addition, POSEIDON groups 1, 2, 3, and 4 had significantly lower AFC than the control group (7.78 ± 0.71, 7.50 ± 0.98, 4.33 ± 0.33, and 4.77 ± 0.34 vs. 12.47 ± 1.73, respectively; Table 1). Furthermore, and as expected POSEIDON Groups 1, 2, 3, and 4 had significantly fewer oocytes retrieved than the control group (4.57 ± 0.89, 5.2 ± 1.06, 2.0 ± 0.26, 2.68 ± 0.51 vs. 13.36 ± 0.76, Table 1). Finally, the control group required significantly fewer units of FSH than POSEIDON group 4 (Table 1). No differences were found between the groups in terms of body mass index, and day 3 FSH and estradiol concentrations. All POSEIDON groups showed a significantly lower FORT compared with the control group (Table 1).

### Blood Serum Results

**Figure 2:**
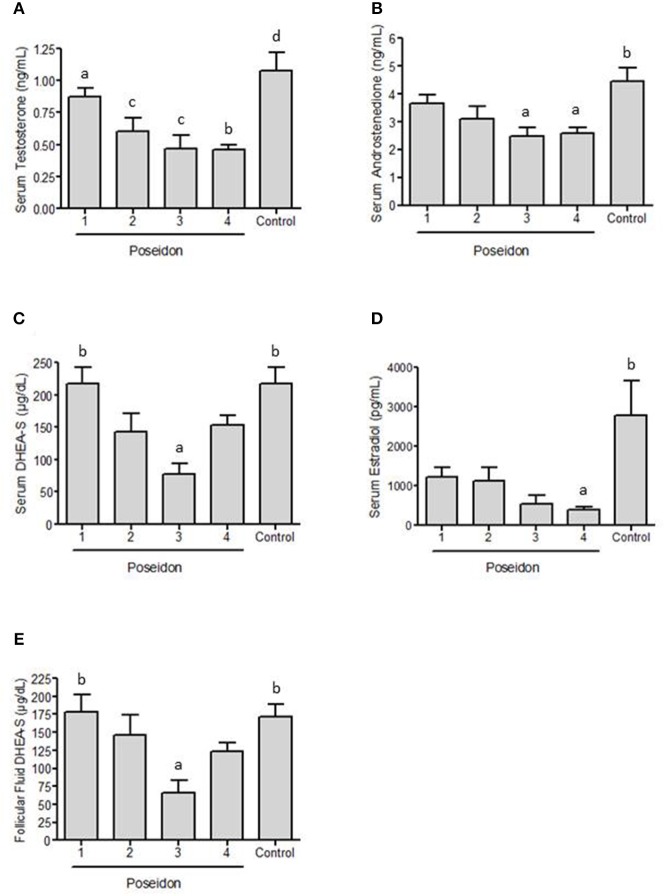
Concentration of hormones in blood serum and follicular fluid of women with poor ovarian response (POR) grouped into POSEIDON categories (1–4) and control women without POR during controlled ovarian stimulation for assisted reproductive technology. Testosterone **(A)**, androstenedione **(B)**, dehydroepiandrosterone sulfate (DHEA-S, **C**) and estradiol **(D)** were evaluated in blood serum. Panel **(E)** shows DHEA-S levels in follicular fluid. a ≠ b, *p* < 0.05; b & c ≠ d, *p* < 0.05.

Figure 2A shows that serum testosterone concentration was significantly higher in controls than in POSEIDON groups 2, 3, and 4 (Table 2). Additionally, POSEIDON group 1 had significantly higher serum testosterone concentration than group 4 (Table 2; Figure 2A).

**Table 2:** Hormonal values in Serum at the aspiration day.

	**Poseidon 1**	**Poseidon 2**	**Poseidon 3**	**Poseidon 4**	**Control**	**ANOVA**
	**(*n* = 14)**	**(*n* = 8)**	**(*n* = 6)**	**(*n* = 22)**	**(*n* = 19)**	***P***
Testosterone (ng/mL)	0.87 ± 0.07^c^	0.61 ± 0.11^a^	0.47 ± 0.10^a^	0.46 ± 0.04^a^	1.08 ± 0.14	0.000
Androstenedione (ng/mL)	3.67 ± 0.29	3.12 ± 0.42	2.49 ± 0.30^a^	2.59 ± 0.21^a^	4.46 ± 0.49	0.001
DHEA-S (μg/dL)	217.57 ± 26.02^b^	143.55 ± 27.37	77.71 ± 16.09^a^	153.10 ± 16.18	218.29 ± 25.30	0.004
Estradiol (pg/mL)	*1, 240*±243	*1, 134*±331	557 ± 210	407 ± 68^a^	*2, 798*±883	0.012
SHBG (nmol/L)	152.05 ± 24.53^c^	144.56 ± 27.8	113.27 ± 27.4	82.73 ± 10.25^a^	161.17 ± 16.59	0.007
IGF-1 (ng/mL)	341.22 ± 36.55	295.82 ± 50.39	338.28 ± 45.25	316.82 ± 40.79	277.89 ± 28.76	NS
FAI	2.74 ± 0.52	1.67 ± 0.30	1.74 ± 0.42	2.33 ± 0.25	2.78 ± 0.42	NS

*Values are expressed as mean ± SEM*.

a *p < 0.05 vs. Control*.

b *p < 0.05 vs. Poseidon 3*.

c *p < 0.05 vs. Poseidon 4*.

*Statistical significance using ANOVA withTukey post-hoc test*.

Regarding androstenedione, Figure 2B shows that patients from the control group had significantly higher serum concentrations than POSEIDON groups 3 and 4 (Table 2). Figure 2C shows that POSEIDON group 3 had significantly lower serum DHEA-S concentrations than controls and POSEIDON Group 1 (Table 2). There was no significant difference between the groups regarding IGF-1 serum concentrations and the free androgen index (FAI) (data not shown). Figure 2D shows that serum estradiol was significantly lower in POSEIDON group 4 compared to the control group (Table 2). In summary we observe that POSEIDON groups 3 and 4 are characterized by low serum levels of testosterone and androstenedione, and POSEIDON group 3 additionally has decreased serum levels of DHEA-S. Additionally, the results show that POSEIDON groups 1 and 2 did not present hypoandrogenemia compared to the control group.

### Follicular Fluid Results

There was no significant difference in follicular fluid concentrations of testosterone, androstenedione, estradiol, IGF-1 and SHBG between the groups. Figure 2E shows that the only significant difference present in follicular fluid was the decreased concentration of DHEA-S in POSEIDON group 3 compared to group 1 and the control group (Table 3).

**Table 3:** Hormonal values in follicular fluid on the aspiration day.

	**Poseidon 1**	**Poseidon 2**	**Poseidon 3**	**Poseidon 4**	**Control**	**ANOVA**
	**(*n* = 14)**	**(*n* = 8)**	**(*n* = 6)**	**(*n* = 22)**	**(*n* = 19)**	***P***
Testosterone (ng/mL)	1.76 ± 0.25	2.12 ± 0.35	2.09 ± 0.76	2.06 ± 0.45	2.34 ± 0.68	*NS*
Androstenedione (ng/mL)	14.41 ± 2.18	20.24 ± 3.78	15.25 ± 7.33	18.95 ± 3.35	25.52 ± 6.22	*NS*
DHEA-S (μg/dL)	178.08 ± 24.37^b^	146.09 ± 28.46	66.43 ± 17.52^a^	123.27 ± 12.11	171.60 ± 16.96	0.009
Estradiol (ng/mL)	468.21 ± 359.79	316.66 ± 294.71	554.62 ± 390.00	251.45 ± 131.52	191.22 ± 171.09	*NS*
SHBG (nmol/L)	83.02 ± 11.67	101.89 ± 29.85	49.05 ± 10.89	49.28 ± 6.19	91.06 ± 12.44	*NS*
IGF-1 (ng/mL)	234.65 ± 29.57	218.99 ± 27.37	229.17 ± 63.08	227.75 ± 24.56	199.46 ± 21.91	*NS*

*Values are expressed as mean ± SEM*.

a *p < 0.05 vs. Control*.

b *p < 0.05 vs. Poseidon 3*.

*Statistical significance using ANOVA withTukey post-hoc test*.

**Figure 3:**
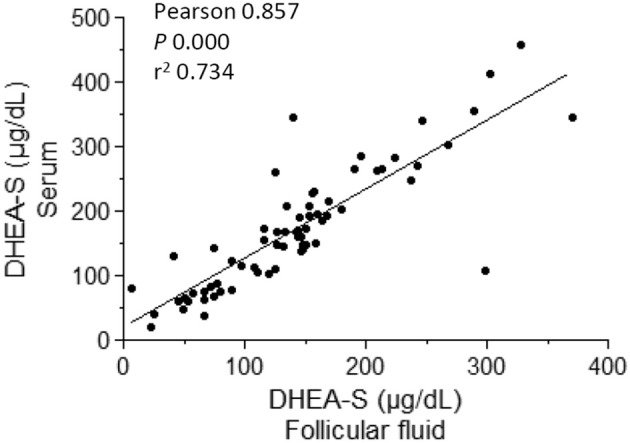
Correlation analysis between blood serum and follicular fluid dehydroepiandrosterone sulfate (DHEA-S) levels in women at oocyte pick-up in assisted reproductive technology.

Since DHEA-S levels were decreased both in FF and serum in POSEIDON group 3 (Table 4), we performed a scatter plot analyzing the correlation between serum and FF DHEA-S in all samples because it has been suggested that serum and follicular fluid testosterone concentrations do not correlate, questioning the potential effect of androgen supplementation to improve the follicular endocrine milieu (14). We observed a high correlation between serum and FF DHEA-S (Figure 3) suggesting that intrafollicular DHEA-S concentration seem to be highly dependent on its circulating levels. No relevant correlations were observed in serum vs. FF for the other analytes studied. The complete dataset with analytes measurements is available in Supplementary Table 1.

**Table 4:** Androgens concentration in follicular Fluid and blood serum on the aspiration day.

**Group**	**FF testosterone**	**Serum testosterone**	**Student *t*-test**	**FF androstenedione**	**Serum androstenedione**	**Student *t*-test**	**FF DHEA-S**	**Serum DHEA-S**	**Student *t*-test**
	**(ng/mL)**	**(ng/mL)**	***P***	**(ng/mL)**	**(ng/mL)**	***P***	**(μg/dL)**	**(μg/dL)**	***P***
Poseidon 1	1.76 ± 0.25	0.87 ± 0.07	0.004	14.41 ± 2.18	3.67 ± 0.29	0.000	178.08 ± 24.37	217.57 ± 26.02	0.278
(*n* = 14)									
Poseidon 2	2.12 ± 0.35	0.61 ± 0.11	0.003	20.24 ± 3.78	3.12 ± 0.42	0.003	146.09 ± 28.46	143.55 ± 27.37	0.950
(*n* = 8)									
Poseidon 3	2.09 ± 0.76	0.47 ± 0.10	0.86	15.25 ± 7.33	2.49 ± 0.30	0.142	66.43 ± 17.52	77.71 ± 16.09	0.646
(*n* = 6)									
Poseidon 4	2.06 ± 0.45	0.46 ± 0.04	0.02	18.95 ± 3.35	2.59 ± 0.21	0.000	123.27 ± 12.11	153.10 ± 16.18	0.148
(*n* = 22)									
Control	2.34 ± 0.68	1.08 ± 0.14	0.84	25.52 ± 6.22	4.46 ± 0.49	0.003	171.60 ± 16.96	218.29 ± 25.30	0.135
(*n* = 19)									

*Values are expressed as mean ± SEM*.

## Discussion

In the present study we analyzed testosterone, androstenedione, DHEA-S, estradiol, SHBG and IGF-1 levels in blood serum and FF from POR women divided into the four categories of the POSEIDON stratification and control women without POR on the day of oocyte retrieval after COH for ART. The blood serum analysis showed that the circulating levels of androgens in women under COH without POR are higher than those during spontaneous cycles. Also we determined that, compared with the control group, the levels of total testosterone, androstenedione and DHEA-S were not different in POSEIDON group 1 but significantly decreased in POSEIDON group 3. Also, levels of DHEA-S in FF were also significantly decreased in POSEIDON group 3 compared with controls. In addition, serum testosterone was decreased in POSEIDON groups 2 and 4 vs. control; and serum androstenedione was reduced in POSEIDON group 4 vs. control.

It is interesting that women in group 1 of the POSEIDON category were the only ones that did not show differences in the measured analytes in blood serum and FF compared with the control group. This particular group is characterized by having ≤35 years old and normal parameters of ovarian reserve which will otherwise would be considered as having normal ovarian function, but their response to COH was poor. In some poor responders, raising the dose of FSH may help to achieve an increase in follicle recruitment. However, a poor response to FSH which results in a low yield of retrieved oocytes cannot be foreseen before starting COH in this group of women. Although the basis for a low response to COH in POSEIDON group 1 is not well-understood, it is known that the expression of the FSH receptor as well as its ability to get activated and transduce the FSH signal seems to be a determinant of gonadotropin treatment (15). Thus, altered FSH receptor expression and/or function could be a factor that may account for the poor ovarian response to COH in POSEIDON group 1.

The only group that presented decreased levels of DHEA-S in serum and FF compared with the control group was POSEIDON group 3. This result was interesting but should be taken with care and as part of a pilot study. If this finding is confirmed with a bigger sample size, several questions may arise from this finding: is the reduced DHEA-S level revealing a distinct etiology for the POR condition respect of the other POSEIDON groups? Will a pre-treatment with DHEA-S benefit particularly women within POSEIDON group 3, improving their ovarian response to COH?

Androgen levels decline with age, as established by Davison *et al*. in a large population-based study in 2005. The authors enrolled over 1,500 women and found that serum testosterone, free testosterone, androstenedione, and DHEA-S declined continuously from 18 to 75 years of age in healthy women, with most of this (78%) occurring during the reproductive years (16).

Gleicher *et al*. demonstrated that both older and younger women with poor ovarian response have significantly lower circulating levels of total testosterone than controls during natural menstrual cycles, even after adjusting for body mass index, age, and race (17). In addition, although it is well-established that under basal conditions women with poor ovarian response present with hypoandrogenemia, it is unknown whether this condition persists under COH during IVF.

Serum levels for DHEA-S in a cohort of 459 women with normal ovarian reserve undergoing their first long protocol for ICSI, positively correlated with the AFC and matured oocyte number but not for clinical pregnancy (OR 1.001, 95% CI, 0.999- 1.004) (18). Nevertheless, in patients with low AMH level (<6.5 pmol/L), DHEA-S appears to be predictive of clinical pregnancy in women of <37.5 years old (19) and live birth (AUC-ROC 0.69, 95% CI 0.59–0.79) (20). Androgen pre-treatment has been considered as an adjuvant in POR patients, although some controversy has been raised about its use. We found that women from POSEIDON group 3 showed significantly different DHEAS levels in serum and follicular fluid. Although the etiology of reduced production of this androgen of adrenal origin is not clear, the administration of DHEA in this group of patients might improve the follicular recruitment during COH.

In a primate animal model, androgens stimulate the growth and survival of small follicles (21) and induce FSH receptors in granulosa cells (22) along with the IGF-1 signaling system in primordial follicle oocytes (9). Interestingly, AR expression has not been detected in primordial follicles. The differences we found in serum testosterone, androstenedione and DHEA-S in some of the POSEIDON groups with respect to the control group suggest that decreased androgens may play a role in POR; however it is not clear whether this is a causative effect or simply an epiphenomenon. The reduced levels of DHEA-S in FF and serum in POSEIDON group 3 are intriguing and whether DHEA has a direct effect on human fertility is still uncertain. While a meta-analysis of six randomized controlled trials (RCT) and 2 observational studies including a total of 745 patients showed that DHEA supplementation had significantly higher implantation and pregnancy rates, as well as significant improvement of ovarian markers (23, 24), not all support such favorable outcomes (25). Such conflicting data regarding the effects of DHEA supplementation in POR women before ART procedures has raised concerns in some authors that had criticized it and deemed it as experimental and non-reliable (26). Nevertheless, in a worldwide survey conducted by IVF physicians from 45 countries, nearly 25% include the use of DHEA in POR patients for COH (27). The discrepancies regarding the benefit of DHEAS treatment before IVF could be related with the fact that not all POR women present with hypoandrogenemia, hence the potential clear advantage from this intervention might be limited to POR women within POSEIDON group 3. Further studies on androgen pre-treatment therapies in ART are required before a particular androgen, dose, and timing scheme can be recommended for each POSEIDON subcategory. Interestingly in a recent study regarding DHEA supplementation before ART in POR women, the group with lower serum DHEA-S concentration (<180 μg/dL) had a 5.92-fold increase in the number of patients with >3 oocytes retrieved compared to those with higher DHEA-S concentration. This finding suggests that DHEA supplementation seems to benefit particularly women with androgen deficiency likely of adrenal origin (28).

The major limitation of the present study is the limited sample size for POSEIDON group 3; however, it is reasonably significant to obtain valuable data on the particular characteristics of this POR subcategory that should be confirmed.

We observed a high correlation between the DHEA-S concentration in serum and FF when pooling the samples from all group from the present study, which is in line with a previous report (29). Interestingly, that correlation was not observed for DHEA suggesting that the desulfated DHEA in FF is subjected to enzymatic metabolism, used as substrate for downstream synthesis of ovarian steroids. Most of the DHEA and DHEAS in serum and follicular fluid are produced by the zona *reticularis* of the adrenal cortex, which with previous desulfonation, can be converted by the granulosa cells to androstenedione and estrone, the precursors for testosterone and estradiol, respectively (30). Interestingly, we found that the DHEAS level in follicular fluid and serum were decreased in women from POSEIDON group 3, however circulating and follicular levels of testosterone and estrogen were not decreased compared to the control group. Although it has been thought that the potential benefits of DHEA are related with its use as a substrate for steroid hormone synthesis, DHEA supplementation in women with diminished ovarian reserve increased the expression of AR in granulosa cells (31). Other potential direct effects on ovarian cells could take place since DHEA may interact directly with certain cytoskeleton components or novel membrane receptors. DHEA was found to bind to microtubule-associated protein (MAP) 2C with strong affinity (32), and a DHEA receptor was found on endothelial cell plasma membranes and it was coupled to endothelial nitric-oxide synthase (eNOS) activity through G_i/o_ proteins Gα_i2_ and Gα_i3_ (33). DHEA(S) may also have actions at other receptors, including the peroxisome proliferator-activated receptor α (PPARα), pregnane X receptor, constitutive androstanol receptor, and estrogen receptor β (34).

In conclusion, in this work we have characterized the levels of androgens, estradiol, sex hormone-binding globulin (SHBG), and insulin-like growth factor 1 (IGF-1), in the serum and FF of women undergoing an IVF cycle categorized in the 4 POSEIDON groups and contrasted with control patients. Our results showed that although hypoandrogenemia was observed in all POSEIDON groups with some groups having more than one androgen significantly decreased with respect to the control group, POSEIDON group 3 particularly had decreased DHEA-S levels both in serum and FF. Such a finding suggests that their POR condition would benefit from a customized therapy to correct such deficiencies, in order to maximize their response to COH for IVF.

## Ethics Statement

In this retrospective cross-sectional study, we evaluated the charts of patients who were treated in a University fertility clinic setting (Maternal and Child Research Institute (IDIMI), University of Chile, Santiago, Chile) between October 2014 and October 2017. Signed informed consent was obtained from all patients enrolled in the study under the approval of the Ethics Review Board from Servicio de Salud Metropolitano Central, Santiago, Chile (September 2013); in accordance with the Declaration of Helsinki. The protocol was approved by the Ethics Review Board from Servicio de Salud Metropolitano Central, Santiago, Chile.

## Author Contributions

The present work was designed by AF, KS, and AT-P. Data extraction and analysis were performed by AM, PC, AG, and AF. Patient recruitment was undertaken by AS, AG, JE, and PC. The initial manuscript draft was prepared by KS and subsequently revised by AF, AT-P, and PC. All the authors approved the final submitted version.

### Conflict of Interest Statement

The authors declare that the research was conducted in the absence of any commercial or financial relationships that could be construed as a potential conflict of interest.

## Supplementary Material

Supplementary Table 1 Total FSH dose, number of days of stimulation and testosterone, adrostenedione and dehydroepiandrosterone sulfate (DHEA-S) levels in follicular fluid and blood serum of each woman included in our study. Subjects have been categorized according to their respective POSIDON group or control group.
